# A Few Other Charities

**Published:** 1894-12-22

**Authors:** 


### A FEW OTHER CHARITIES.

**British Home for Incurables**.—Helpless, hopeless, homeless. The Board of Management of the British Home for Incurables earnestly appeal for 500 new annual subscribers of one guinea each, to counterbalance the loss of income sustained by selling out capital to build the new home at Streatham. In addition to maintaining the suffering inmates of the present home, the charity has nearly 300 pensioners, each receiving £20 a-year. Applications from all parts of the United Kingdom are constantly being received. Cheques and postal orders to be crossed "Barclay and Co.," and sent to Mr. R. G. Salmond, Secretary, 73, Cheapside, E.C.

**City of London Truss Society**, 35, Finsbury Square.—For the relief of the ruptured poor throughout the kingdom. At the present time nearly 10,000 of both sexes and all ages are treated annually, while nearly half a-million patients have been relieved since its formation eighty-seven years ago. The recently enlarged premises provide separate entrances for male and female patients; and there is a female attendant for the latter. Secretary, Mr. John Whittington.

**London Orphan Asylum, Watford**.—The managers appeal for extended support in aid of this old-established and national charity. Dispensing relief without distinction of trade, profession, or locality, the committee feel justified in asking for commensurately general support. New annual subscriptions are much needed. £14,000 is required each year to carry on the work. The number of candidates, and the urgency of the "last time" cases, coincident with a large number of vacancies, have induced the managers to give relief to forty-five children at the next election, and they earnestly hope that their action will commend itself, and evoke support from the charitable public. Subscriptions will be thankfully received by the secretary at the office, 21, Great St. Helen's, E.C.

**Metropolitan Drinking Fountain and Cattle Trough Association**.—This is the only society which provides free supplies of water for the many thousands of men, women, and children, besides horses, cattle, sheep, and dogs that are daily toiling through our streets. It depends entirely on voluntary contributions, which are urgently needed. Secretary, Mr. M. W. Milton, 70, Victoria Street, Westminster, S.W.

**Orphan Working School**, Haverstock Hill and Hornsey. Offices, 73, Cheapside, E.C. Founded 1758.—This is a national and undenominational institution which maintains 500 children, varying in age from infancy to fourteen or (in special cases) fifteen years. It is greatly in want of funds at the present time, and £2,000 is urgently needed to meet immediate and pressing liabilities. This orphanage is the oldest one of the kind in the metropolis, and over 90 per cent. of the old scholars turn out satisfactory. Secretary, Algernon C. P. Coote, M.A.

